# Pathogenesis of Pachyvein Formation in Central Serous Chorioretinopathy: A Hydrodynamic Analysis

**DOI:** 10.3390/jcm13164777

**Published:** 2024-08-14

**Authors:** Okihiro Nishi, Yutaro Nishi, Miki Tatsumichi, Tsutomu Yasukawa

**Affiliations:** 1 Jinshikai Medical Foundation, Nishi Eye Hospital, 4-14-26 Nakamichi, Higashinari-ku, Osaka 537-0025, Japan; y.nishi@nishi-ganka.or.jp (Y.N.); m.tatsumichi@nishi-ganka.or.jp (M.T.); 2 Department of Ophthalmology and Visual Science, Nagoya City University Graduate School of Medical Sciences, Nagoya 464-8601, Japan; yasukawa@med.nagoya-cu.ac.jp

**Keywords:** central serous chorioretinopathy, pachyveins, laser speckle flowgraphy, fluid mechanics

## Abstract

**Objectives:** To attain an understanding of pachyvein formation seen with central serous chorioretinopathy (CSC) by measuring the choroidal blood flow (CBF) velocity and to apply the findings to existing hydrodynamic theorems. **Methods:** Nineteen subjects with monocular CSC (mean age 51.8 ± 12.7 years) were included. Laser speckle flowgraphy measured the mean blur rate (MBR) in the foveal and perifoveal regions, and the findings were applied to the Equation of Continuity (Q = AV) and Bernoulli’s theorem (1/2V^2^ + P/ρ = constant). **Results:** The mean foveal/perifoveal MBRs in the CSC eyes were 6.4 ± 3.2/9.6 ± 3.2 (*p* < 0.001). The mean MBRs in the non-CSC eyes were 8.3 ± 3.2/7.9 ± 2.4 (*p* = 0.37). The significant foveal CBF velocity decrease in CSC eyes occurs because of exudation from the hyperpermeable choriocapillaris. The subsequent decrease in blood volume due to the exudation elicits a decrease in the blood flow velocity in the inferior venules in accordance with the Equation of Continuity. The decrease in the blood flow velocity may result in an elevated blood flow pressure in the inferior venules and veins at the exudation site, in accordance with Bernoulli’s theorem. **Conclusion:** A significant decrease in the foveal MBR may result from exudation from the hyperpermeable macular choriocapillaris. This decreased velocity may elicit a blood pressure elevation that can expand the inferior venules and veins at the exudation site, so-called pachyvein and pachychoroid formation. The application of hydrodynamic theorems to the measurement of ocular blood flow can provide new insights into the understanding of the pathogenesis of chorioretinal disease.

## 1. Introduction

Pachychoroid spectrum diseases [1] include a variety of clinical findings such as central serous chorioretinopathy (CSC) [1,2,3], choroidal vascular hyperpermeability [4], late choroidal dye filling on indocyanine green angiography (ICGA) [4], pachychoroid [3], pachyveins [5], anastomosis in vortex veins [4,6], thickened sclera [7], loculation of fluid in the suprachoroid [8,9], and pigmentepitheliopathy [10].

Some theories of the pathogenesis of pachyveins and pachychoroid formation, clinical hallmarks of CSC, have been proposed. Kishi et al. [4] reported areas of filling delay in the choriocapillaris and dilation of the emissary vortex vein resulting from congestion of the dominant vortex veins. Imanaga et al. [7] postulated that scleral thickening may choke the emissary vortex vein.

We reported that pachyveins and pachychoroid formation results from increased blood pressure that causes an expansion of choroidal venules and veins [11,12]. We used two theorems of hydrodynamics [13] to clarify these clinical entities.

The first theorem is the Equation of Continuity, Q = AV, in which Q represents the blood flow volume; A, the sectional area of a vessel; and V, the blood flow velocity. The Equation of Continuity states that decreased blood flow volume occurs simultaneously with decreased blood flow velocity, and the reverse is true as well.

The second theorem is Bernoulli’s principle, 1/2V^2^ + P/ρ = constant, in which V represents the blood flow velocity; P, the static pressure; and ρ, the blood density. Bernoulli’s principle states that the decreased velocity of a fluid occurs simultaneously with increased static pressure, and the reverse is also true.

By applying these theorems, we attempted to explain the pathogenesis of pachyveins, and specifically, that the hyperpermeability of the choriocapillaris in the foveal region, as visualized on ICGA, results in exudation that causes decreased blood flow volume Q in the inferior choriocapillaris, venules, and veins. In accordance with the Equation of Continuity, this decreased blood flow volume occurs simultaneously with decreased blood flow velocity V, seen on ICGA, as a filling delay in the choriocapillaris [4].

In accordance with Bernoulli’s principle, decreased blood flow velocity occurs simultaneously with increased static blood pressure P within the venules and veins, causing the venules and veins to expand and resulting in pachyvein formation [11,12]. When this venous expansion exceeds the physical limit at the scleral foramen, through which the vortex vein traverses, vortex vein compression results in blood flow congestion, further enhancing pachyvein formation.

In our previous report, the above-mentioned decrease in choroidal blood flow (CBF) velocity was based on a simple observational clinical finding, namely, a delay in the filling of the choriocapillaris seen on ICGA [4]. We interpreted the filling delay of dye as a slowing of the blood flow velocity. Since the CBF velocity was not measured in the previous study, we measured the CBF velocity in the foveal and perifoveal regions in CSC eyes using laser speckle flowgraphy (LSFG).

## 2. Materials and Methods

The ethics committee of Nishi Eye Hospital approved this prospective observational case series study, which followed the tenets of the Declaration of Helsinki.

### 2.1. Patients and Examinations

Nineteen eyes of nineteen treatment-naïve subjects (3 women [15.8%] and 16 men [84.2%]), seen between July 2022 and March 2023, were studied. The patient ages ranged from 36 to 79 years (mean, 51.8 ± 12.7 years).

The inclusion criteria included the diagnosis of acute monocular CSC. Each subject presented with acute monocular CSC. The fellow non-CSC-affected eye had no exudation from the choriocapillaris seen on fluorescein angiography (FA) and ICGA and served as the control. Fellow eyes with exudation from the choriocapillaris with or without central serous edema were excluded. The patients who had undergone any previous surgery and had other diseases also were excluded.

### 2.2. FA, ICGA, and Optical Coherence Tomography (OCT)

**Figure 1:**
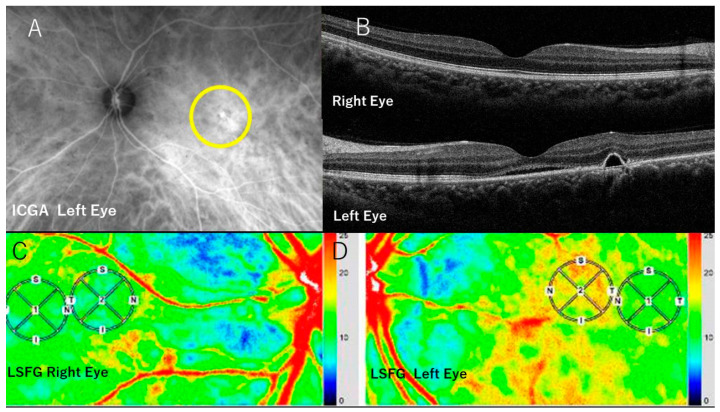
Photographs of the case 1. (**A**) ICGA shows choriocapillaris hyperpermeability in the macular region, with ICG dye leakage within the circle. (**B**) Horizontal section on OCT in the left CSC eye with pachyveins (below) and the unaffected right eye. (**C**) LSFG in the right unaffected eye. The color bar at the right margin indicates the blood flow velocity. The velocity is faster toward red and slower toward blue. The blood flow velocity in the foveal region (in circle ①) reflects a CBF signal and is only slightly lower compared to an adjacent area (in circle ②), a finding that may mimic the physiological condition. (**D**) LSFG in the left CSC eye. Note that the blood flow velocity in the area with choroidal vasculature hyperpermeability in A involving the foveal region (within the circle ①) is extremely slow (indicated by green), compared to that in the surrounding area (in circle ②). This indicates a large decrease in blood flow velocity in the area affected by CSC, suggesting, in accordance with Bernoulli’s theorem, a blood pressure rise in the inferior zones of circulation.

Both eyes of 19 subjects underwent FA, ICGA (Figure 1A) and OCT (swept source OCT, Topcon, Tokyo, Japan). The choroidal thickness was measured (Figure 1B) at the fovea by pointing at the lower end of the pigment epithelium layer and that of the choroid manually on the OCT image. One author (M.T.) performed all measurements and evaluations. The distance between two points was calculated automatically.

### 2.3. LSFG

LSFG (Softcare, Fukuoka, Japan) measured the CBF velocity, the so-called mean blur rate (MBR), a quantitative index of blood flow velocity and blood volume [14,15,16]. An 830 nm wavelength diode laser was applied to the posterior pole at the macular region. The reflected light from the plane of the lasered tissue formed a speckle pattern caused by the movement of erythrocytes that produced a blurred image within this speckle pattern. The MBR was calculated from variations within this blurred region that provided an index of the relative flow velocity.

Within each obtained image and within a 1 mm circle (circle ①) in which CSC was present, the velocity of the CBF (the MBR) at the foveal point was measured (foveal MBR). LSFG provided the foveal point automatically by default. After obtaining the LSFG figures from all CSC eyes, we first noted that the foveal point with a low MBR was surrounded incompletely by the perifoveal area with a higher MBR. Within this perifoveal region with the higher MBR, a 1 mm circle (circle ②) was arbitrarily drawn, adjoining circle ① and avoiding retinal vessels (perifoveal MBR) (Figure 1C,D). Similar measurements were performed in the contralateral (control) CSC-unaffected eyes. The foveal point was determined automatically by default, and circle ② by superimposing circle ② of the CSC eye.

### 2.4. Statistical Analysis

The Wilcoxon signed-rank sum test was used for the choroidal thickness and all comparisons of MBR values in the fovea and perifovea, and for the differences in the MBR between eyes with and without CSC.

### 2.5. Simulated Blood Pressure Calculation in the Choroidal Venules during Choriocapillaris Exudation

The static pressure, defined as the blood pressure in the choroidal venules and veins during exudation from the choriocapillaris in the macular region of CSC eyes, was calculated and compared with the blood pressure in the normal physiological state without exudation. In the simulated setting, preliminary LSFG data were obtained and applied to Bernoulli’s theorem.

For the venules in the normal physiological state, the following equation was applied: 1/2V_1_^2^ + P_1_/ρ = Constant_1_ (C_1_), where V_1_ and P_1_ represent the blood flow velocity and blood pressure, respectively, in the venules.

At the site of the venules inferior to the choriocapillaris in which exudation occurs, the following equation was used: 1/2V_2_^2^ + P_2_/ρ = Constant_2_ (C_2_), where V_2_ and P_2_ represent the blood flow velocity and blood pressure, respectively, in the venules.

When exudation from the choriocapillaris occurred, the kinetic energy of the blood flow lessens in the inferior venous fields, and C_2_ is smaller than C_1_. Thus, the equation should read as follows: 1/2V_1_^2^ + P_1_/ρ > 1/2V_2_^2^ + P_2_/ρ.

To obtain the simulated blood pressure value in the venules during the exudation in the choriocapillaris, the following values were read or calculated.

1. The flow velocity V_1_ and flow pressure P_1_, respectively, in the venules, in the physiological condition [17].
2. Since the MBR value is proportional to the blood flow velocity [12,13,14], the unit of the MBR value for the blood flow velocity is converted into mm/s using the mean macular MBR of the non-CSC eyes and V_1_.
3. Calculation and conversion of the MBR value of V_2_ into mm/s.
4. Calculation of P_2_ by applying the V_1_, V_2_, and P_1_ values to Bernoulli’s theorem.
5. The P_2_ value depends on the degree and extent of energy loss, i.e., exudation in the choriocapillaris. The energy loss can be expressed as the percentage of C_1_, i.e., C_2_ = C_1_ − nC_1_: n = percentage of energy loss. The C_1_ value is calculated by substituting V_1_ and P_1_ values. The relationship between P_2_ and C_2_ is then established with the varying n values.

## 3. Results

Table 1 summarizes the results.

ICGA and OCT: All CSC eyes had a central serous detachment and ICG dye leakage in the macular region (Figure 1A, B).

### 3.1. Choroidal Thickness

The choroidal thicknesses in the CSC eyes ranged from 203 to 614 μm (mean. 402.2 ± 92.4 μm), and in the non-CSC fellow eyes, from 165 to 571 μm (mean. 331.7 ± 92.7 μm), a difference that reaches significance (*p* < 0.001).

### 3.2. MBRs in the CSC Eyes

The MBRs in the foveal point ranged from 0.9 to 12.8 (mean. 6.4 ± 3.2). The MBRs in the perifoveal point ranged from 5.2 to 16.6 (mean. 9.6 ± 3.2) (*p* < 0.001), a difference that reached significance (Table 1). Thus, the CSC eyes had a foveal choroidal point at which the blood flow velocities were significantly lower compared to the perifoveal point, at which the blood flow velocities were significantly higher (Figure 1D).

The differences between the foveal MBR and perifoveal MBR ranged from −6.1 to −1.0 (mean. −3.1 ± 1.4) (Table 1).

In cases 10, 12, 15, and 19, the foveal MBRs were slightly higher with very small differences compared with the unaffected control eyes. In cases 3, 4, 9, 13, 14, 17, and 18, the perifoveal MBRs were lower than those of the control eyes.

### 3.3. MBRs of the Unaffected Contralateral Eyes

In the unaffected contralateral eyes (Figure 1C), the MBRs in the foveal region ranged from +3.4 to +13.1 (mean. 8.3 ± 3.2). The MBRs in the perifoveal regions ranged from +3.0 to 11.0 (mean. 7.9 ± 2.4). The difference did not reach significance between the two mean MBRs (*p* = 0.37).

The differences between the MBRs in the foveal and perifoveal regions ranged from −2.1 to +4.4 (mean. 0.4 ± 1.4).

Significant differences were seen in the foveal MBRs (*p* = 0.017) and the perifoveal MBRs (*p* = 0.033) between the CSC eyes and non-CSC eyes.

The MBR difference between the foveal and perifoveal regions in the CSC eyes was significantly greater than in the non-CSC eyes (*p* < 0.001).

### 3.4. Simulated Blood Pressure Calculation in the Choroidal Venules during Exudation from the Choriocapillaris

When the mean MBR values of 9.6 MBR and 6.4 in the CSC eyes (Table 1) were used for V_1_ and V_2_, respectively, the ratio between V_1_ and V_2_ was 0.66 V_1_ = V_2_, equating to a 34% decrease in the blood flow velocity in the venules in the macular region.

1. The flow velocity V_1_ and pressure P_1_ values in the venules in physiological conditions were 1 cm/s = 10 mm/s and 18 mmHg, respectively [17].
2. The mean foveal MBR of the non-CSC eyes was 8.3 (Table 1), and the flow velocity in the venules V_1_ was 10 mm/s. Therefore, 1 MBR = 10/8.3 mm/s = 1.2 mm/s.
3. V_2_ = 6.4 mean foveal MBR × 1.2 mm/s = 7.7 mm/s.
4. $P_{2}<P_{1}+\frac{\rho }{2}\left({V_{1}}^{2}-{V_{2}}^{2}\right)=18+\frac{\rho }{2}\left(10^{2}-7.7^{2}\right)$ = 8 + 21 = 39 mmHg (calculations in Table 2).
5. C_1_ = 67.1, when 10 mm/s and 18 mmHg for V_1_ and P_1_, respectively, are substituted into the formula. The results are shown in Table 3.

In the case of a simulated blood pressure setting of P_1_ = 18 mmHg, a flow velocity of V_1_ = 10 mm/s in the venules, and a decreased flow velocity of V_2_ = 7.7 mm/s, the blood pressure P_2_ could reach 39 mmHg, which is a 216% increase (Table 2), provided that there was no exudation, and thus no energy loss.

## 4. Discussion

The mean age and gender distribution of the patients with CSC in the current study were comparable to those reported in a similar Japanese cohort [18]. The mean choroidal thickness was significantly greater in the CSC eyes, similar to the previous reports [1,2,3,4,5,6,7,8].

In the CSC eyes, the MBR on LSFG was significantly lower at the foveal point compared to that at the perifoveal point. In the unaffected fellow eyes that served as controls, no difference in the MBR was seen between these points (Table 1, Figure 1). In the CSC eyes, the choroidal and retinal pigment epithelium changes are often present in the contralateral fellow eye serving as the control. However, the CSC eyes had serous subretinal fluid due to a hyperpermeable choriocapillaris with FA and ICG dye leakage. These cardinal changes were not seen in the fellow eyes. Because the current study focused on the aftermath of hyperpermeable choriocapillaris followed by exudation from it, we think the fellow eyes in our study can be regarded as controls.

These findings suggest that in the CSC eyes, the areas adjacent to the foveal region have a significantly higher MBR compared to the foveal point that has a significantly lower MBR (Figure 1D). Thus, in the CSC eyes, a significant decrease in the CBF velocity was seen in the foveal region—an area in which serous retinal detachments are present—in contrast to adjacent perifoveal areas.

As shown in Table 1, in cases 10, 12, 15, and 19, the foveal MBR was slightly higher with a very small difference from that of the unaffected control eyes. However, in those cases, the perifoveal MBR was high, so the difference was significant. The significantly higher MBR in the perifoveal region contributed to the significant difference.

In cases 3, 4, 9, 13, 14, 17, and 18, the perifoveal MBR was lower than that of the control eyes. However, in those cases, the foveal MBR was markedly low, which contributed to the significant difference.

These findings indicate that the blood velocity difference between the foveal and perifoveal MBRs stems not only from the perifoveal velocity increase but also from the foveal velocity decrease. Thus, the relative decrease in the blood flow velocity matters.

Saito et al. [19,20] reported similar findings in CSC eyes, i.e., high MBR values in the foveal and perifoveal areas that decreased with the regression of CSC. The authors concluded that the pathogenesis of CSC stems from CBF elevation and an imbalance in the distribution of the CBF. However, they did not mention a significant decrease in the foveal blood flow velocity and its hydrodynamic significance.

The current findings suggested that a region with high perifoveal MBR is a consistent finding in the presence of choroidal arterioles and arteries, since this perifoveal region showed the highest MBR (“red” in Figure 1D) in the entire image. A foveal point with a low MBR is indicative of leaking choroidal capillaries with exudation, venules, and veins. Based on this interpretation of the LSFG figures with the corresponding MBRs and the physiological condition of the blood flow sequence [17], the simulated blood pressure in the choroidal venules during the exudation from the choriocapillaris was hydrodynamically calculated (Table 2).

A high perifoveal MBR finding in CSC eyes may result from hyperemia due to inflammation within the perifovea. In hyperemia [21], local vasodilatation occurs due to a histamine and nitric oxide response to oxygen debt. In addition, the accumulation of metabolic waste products also plays a role.

Such a vasodilatory response (enlargement of the sectional area A of a vessel in the Equation of Continuity Q = AV) elicits a simultaneous blood volume increase that coincides with the high perifoveal MBR shown in our findings and coincides with the finding that the MBR difference between the foveal and perifoveal regions in the CSC eyes was significantly greater than that in the non-CSC eyes (*p* < 0.001).

Alternatively, consistent with the Equation of Continuity, a significantly low foveal MBR and a decrease in the CBF velocity occurs because of decreased blood flow volume caused by exudation from a hyperpermeable choriocapillaris. Thus, an increase in the blood flow volume due to arterial inflammatory vasodilatation and a decrease in the blood flow volume in the venules due to exudation in the choriocapillaris contributed to the significant difference between the perifoveal MBR, i.e., the arterial flow velocity and the foveal MBR, i.e., the choriocapillaris and venous flow velocity.

This finding coincides with that of Saito et al. [19,20], who referred to this as an “imbalanced distribution of CBF”. Such an imbalanced distribution of choroidal blood flow was not noted in the non-CSC eyes.

We interpreted the filling delay seen on ICGA in the choriocapillaris to be the result of decreased blood flow velocity in the foveal region [11,12]. The current findings support this hypothesis.

Bernoulli’s theorem states that when a significant decrease in blood flow velocity in the choriocapillaris in the foveal region is present, the static pressure, i.e., the venous blood pressure in the inferior region of the choriocapillaris, venules, and veins, simultaneously increases.

A simulation of Bernoulli’s theorem demonstrated (Table 2) that, with a normal blood pressure of 18 mmHg in the venules, a pressure increase approaching 39 mmHg, an increase of 216%, could have been attained within the capillaries and venules when the mean foveal and perifoveal MBRs were 6.4 ± 3.2 and 9.6 ± 3.2, respectively. Such an increase in blood pressure facilitates the expansion of the choroidal venules and veins, resulting in pachyveins and pachychoroid formation. When the expansion of the veins exceeds the anatomic limit of the scleral foramen aperture through which the vortex vein must traverse, congestion from the constriction of the vortex vein occurs and further enhances pachyvein formation. The actual measurement of the blood pressure in the Sattler and Haller veins and vortex veins awaits future assessment.

Regarding the application of both the Equation of Continuity and Bernoulli’s theorem, which stem from the principle of conservation of energy and mass, they may not be valid in an exudating vessel. With exudation, the weight of the blood decreases with the loss of the kinetic energy that the blood flow possesses, making Bernoulli’s theorem not applicable. However, although there is a certain amount of energy loss due to the exudation from the choriocapillaris, the blood flow with reduced energy may soon become steady in the lower reaches of the venules, because there is no longer further energy loss in that part of the vessel. The steady blood flow, despite the slightly reduced energy, may comply with Bernoulli’s theorem under different conditions than those before the exudation. Because the energy loss could not be determined, it was expressed as the percentage of C_1_ in Table 3.

Based on the above consideration, Bernoulli’s theorem was changed as C_1_ > C_2_ (Table 2) in the mathematical and physical calculation in the simulation. Moreover, the blood pressure was compared in the simulation between that before (normal) and that during exudation within the same venules, whereby the hyperpermeable choriocapillaris site was not included, justifying the application of the theorem.

Table 3 shows in the simulation, when the velocity V_2_ is 7.7 mm/s, P_2_ becomes 17 mmHg, a level similar to the normal pressure P_1_ (18 mmHg), firstly when C_2_ has a 30% energy loss of C_1_. Such a huge energy loss resulting from the exudation of a small amount of blood plasma from the choriocapillaris appears to be inconceivable. Table 3 also shows that even though there is a 10% loss in C_1_, C_2_ becomes 60.4, and P_2_ 35.3 mmHg, still double the original pressure of 18 mmHg, and the lower the energy loss, the greater the pressure elevation, provided that V_1_ = 10 mm and V_2_ = 7.7 mm/s; thus, this is a 23% decrease in the normal velocity.

The above consideration suggests that there is a discrepancy between the dimensions of the energy loss and the velocity decrease measured. In other words, the velocity decrease may be too large in contrast to the dimensions of the energy loss caused by exudation. These considerations suggest that after the exudation from the choriocapillaris, when the blood flow becomes steady without further energy loss, there should be a factor that may contribute significantly to the further decrease in the already decreased blood flow velocity in the venules, resulting in V_2_ = 7.7 mm/s. The blood density ρ after the exudation from the choriocapillaris will increase in the venules due to the blood condensation there because of exudation. In accordance with Bernoulli’s theorem, P_2_ will increase, since P_2_ = ρ (C_2_ − 1/2V_2_^2^). We think this density increase after the exudation from the choriocapillaris plays a crucial role in the increased venous blood pressure and the resultant pachyvein formation.

A greater blood density decreases the blood flow velocity further after the flow velocity decrease due to exudation from the choriocapillaris. This situation is comparable to dehydration syndrome, in which the capillary blood flow slows from the normal 2 s to 3 to 3.5 s, known as the capillary refilling time due to blood condensation by dehydration [22]. Thus, this further velocity decrease in the venules can cause a blood pressure rise there in accordance with Bernoulli’s theorem.

In summing up the simulated results, because the decreased blood flow velocity at the choriocapillaris is accompanied by energy loss resulting from the exudation, Bernoulli’s theorem cannot be applied here. However, in the lower reaches of the exudation site, i.e., venules and veins, the blood flows steadily and there is no longer further energy loss. Thus, Bernoulli’s theorem is valid and applicable in the lower reaches of the exudation site under mass conservation. In the venules, the blood velocity further decreases as a result of the increased blood density ρ due to the blood condensation, in compliance with Bernoulli’s theorem. Therefore, the decrease in the foveal mean MBR (blood flow velocity) measured may be due to exudation from the choriocapillaris, where Bernoulli’s theorem cannot be applied, and the further decrease is due to the increased blood density, where the theorem can be applied.

The limitation of this study is that the sample size may be too small to generalize the theory. Some issues are based on theoretical considerations, for example, the blood condensation in the venules in the wake of exudation and its effect on the flow velocity. The further decrease in the blood flow velocity due to the blood condensation in the venules following the velocity decrease due to the exudation from the choriocapillaris cannot be exactly determined. This was assumed by calculation using mathematical and physical theorems, although the actual reduced MBR data in the macular lesions, which were measured by LSFG, were significantly great enough to suggest a further reduction over and above the exudation in the downstream venous blood flow. The blood pressure elevation in the venules and veins in the wake of the exudation from the choriocapillaris cannot actually be measured. The actual measurement of the blood pressure in the corresponding veins may corroborate the results and awaits future assessment.

In conclusion, we showed that, using LSFG, the foveal choroidal region in CSC eyes displays a significant decrease in blood flow velocity compared with that in the perifoveal sites. The significant velocity decrease is a consequence of exudation from the hyperpermeable macular choriocapillaris, which is in accordance with the hydrodynamic Equation of Continuity theorem. The already decreased blood flow velocity decreases further due to the increased blood density in the lower reaches of venules following the exudation from the choriocapillaris. In accordance with Bernoulli’s theorem, this further velocity decrease may simultaneously elicit a blood pressure rise in the inferior region of the choriocapillaris venules. Such a blood pressure rise may induce expansion in the venules and vortex veins in the Sattler and Haller layers and formation of pachyveins. When the expansion exceeds the diameter of the scleral foramen through which the vortex vein must traverse, reinforced venous congestion results. The actual measurement of blood pressure in the venules and veins in the Sattler and Haller layers may clarify this theoretical but hypothetical consideration.

Finally, the application of hydrodynamic theorems to ocular blood flow can provide new insights into the understanding of the pathogenesis of chorioretinal diseases.

## Figures and Tables

**Table 1:** Choroidal Thickness and Blood Flow Velocity (MBR).

Case		Age	Sex	Choroidal Thickness		MBR (CSC-Eyes)		MBR (Contralateral Eyes)			
				CSC-Eyes	Contra-Lateral Eyes	Fovea	Peri-Fovea	MBR-Difference	Fovea	Peri-Fovea	MBR-Difference
1	H.T.	36	M	310μm	314μm	12.8	16.6	−3.8	10.9	10.4	+0.5
2	Y.K.	55	M	431	324	7.7	11.3	−3.6	7.8	8.8	−1.0
3	T.S.	52	M	343	334	6.0	9.9	−3.9	10.3	11.0	−0.7
4	S.S.	51	M	435	339	3.2	7.0	−3.8	9.6	10.4	−0.8
5	H.N.	38	M	348	245	8.2	9.6	−1.4	8.9	8.4	+0.5
6	Y.I.	35	M	454	367	11.6	13.8	−2.2	12.0	9.0	+3.0
7	H.M.	59	F	505	415	11.5	12.5	−1.0	13.1	8.7	+4.4
8	A.Y.	43	M	322	241	7.2	13.8	−5.6	9.7	8.6	+1.1
9	Y.Y.	67	M	325	312	0.9	5.2	−4.3	3.5	5.6	−2.1
10	T.M.	44	M	203	168	4.5	6.9	−2.4	3.4	3.7	−0.3
11	H.G.	58	M	458	399	3.4	7.0	−3.6	6.2	6.0	+0.2
12	K.Y.	52	F	320	276	4.2	7.1	−2.9	3.6	3.0	+0.6
13	R.H.	83	M	357	165	4.4	7.0	−2.6	10.5	8.0	+2.5
14	K.I.	41	M	415	328	7.7	9.0	−1.3	11.2	9.5	+2.0
15	M.F.	73	F	614	571	3.9	10.0	−6.1	3.6	5.1	−1.5
16	Y.F.	50	M	397	340	4.8	7.4	−2.6	4.4	5.2	−0.8
17	K.K.	45	M	495	409	5.9	7.3	−1.4	10.9	9.8	+1.1
18	H.G.	59	M	443	380	4.7	6.7	−2.0	9.5	9.5	0
19	K.T.	44	M	467	376	9.6	13.9	−4.3	8.7	8.6	+0.1
		51.8 ± 12.7		402.2 ± 92.4	331.7 ± 92.7	6.4 ± 3.2	9.6 ± 3.2	−3.1 ± 1.4	8.3 ± 3.2	7.9 ± 2.4	0.4 ± 1.4
				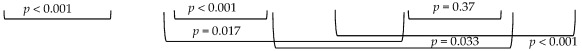							

**Table 2:** Simulated calculation of the blood pressure in the choroidal venules during exudation from the choriocapillaris.

$\frac{1}{2}{V_{1}}^{2}+\frac{P_{1}}{\rho }=C_{1}\left(normal\;state\;in\;venules\right)\;\frac{1}{2}{V_{2}}^{2}+\frac{P_{2}}{\rho }=C_{2}\;(after\;exudation)$	
$C_{1}>C_{2}\;hence,\;\frac{1}{2}{V_{1}}^{2}+\frac{P_{1}}{\rho }>\frac{1}{2}{V_{2}}^{2}+\frac{P_{2}}{\rho }\to P_{2}<P_{1}+\frac{\rho }{2}\left({V_{1}}^{2}-{V_{2}}^{2}\right)$	
V_1_	Blood flow velocity of venules in normal state: 10 mm/s
P_1_	Blood pressure of venules in normal state: 18 mmHg
V_2_	Blood flow velocity of venules during exudation (6.4 mean macular MBR in CSC eyes): see the calculation below.
P_2_	Blood pressure of venules during exudation: the pressure looked for.
ρ	Blood density: 1.05
The unit of MBR value for blood flow velocity is converted into mm/s using the mean macular MBR of the non-CSC eyes, which is 8.3 MBR, corresponding to 10 mm for V_1_: 1 MBR = 10/8.3 mm/s = 1.2 mm/s	
Hence, V_2_ = 6.4 mean macular MBR = 6.4 × 1.2 mm/s = 7.7 mm/s	
$P_{2}<P_{1}+\frac{\rho }{2}\left({V_{1}}^{2}-{V_{2}}^{2}\right)=18+\frac{\rho }{2}\left(10^{2}-7.7^{2}\right)=18+21=39\;mmHg$	
Thus, in simulation, there would be a nearly 216% blood pressure rise from 18 mmHg to 39 mmHg according to Bernoulli’s theorem, when the velocity decreased from 10 mm/sec to 7.7 mm/s.	

**Table 3:** Relationship between energy loss and P_2_ in simulation when the velocity V_2_ is 7.7 mm/s.

Energy Loss (%)	C_2_	P_2_ (mmHg)
(=100 × (C_1_ − C_2_)/C_1_)		
0	67.1	39.4
5	63.6	38.2
10	60.4	35.3
15	57.0	27
20	53.7	23.7
30	47.0	17
40	40.0	10.5
50	34.0	4.2

The energy loss in C_1_ (67.1 in physiological conditions) by exudation is not measurable in vivo. Therefore, it is expressed here in percentages from 0 to 50 (left column) with the corresponding value of C_2_ (energy level after energy loss in C_1_, middle column). P_2_ then can be calculated from Bernoulli’s equation, since C_2_ and V_2_ (7.7 mm/s) are known (right column).

## Data Availability

The original contributions presented in the study are included in the article, further inquiries can be directed to the corresponding author/s.
